# Progressive Paralysis of the Insane*General Paresis, or Incomplete Progressive Paralysis. By John P. Gray, M.D., of Utica. Art. XI. Trans. Med. Soc. of State of N.Y., for 1866.

**Published:** 1867-08

**Authors:** 


### PROGRESSIVE PARALYSIS OF THE INSANE.*

With some show of reason, alienists, who have made the different forms of insanity a life-long study, complain of the inattention of general practitioners to the recent discoveries in the pathology of the nervous system. They say the larger number of cases affected with the paralysis peculiar to the insane, who enter our asylums for treatment, have either been improperly diagnosed or the patient's friends have been deluded by an unjustifiable prognosis of recovery. And it becomes the duty of family physicians to notice the change, for every superintendent of an insane institution states that the cases do progress unfavorably after their admission, while there is good reason to believe much good could be done medicinally in the incipiency of the attack. Although the affection has been noticed for a long time, and, twenty years ago, Dr. Pliny Earle† published the first full account of the disease in this country, yet Flint speaks of general paralysis simply as general spinal paralysis, *i.e.*, when the four extremities are affected, and Aitken discusses in four lines our subject. But the excuse for this omission is not the rare occurrence of the disease. An examination of the abstracts of the reports from our insane asylums, in the *Am. Jour. Med. Sci.*, for the last fifteen months, shows that among 14,566 cases of insanity in thirty-four institutions, there were 1159 deaths, and in twenty-five of the institutions, which report the causes of death, there were 72 deaths from general paralysis or paresis.

In Dr. Gray's essay, we have an able description of this disease. In the incipiency of the disorder, the attention of the physician should be drawn to the mental alienation by the extravagance of ideas. Generally, a man who has transacted a moderate business will be found making large contracts and embarking in new enterprises without any reference to his capital. His delusion is expressed by his often repeating, "I

* General Paresis, or Incomplete Progressive Paralysis. By John P. Gray, M.D., of Utica. Art. XI. Trans. Med. Soc. of State of N.Y., for 1866.

† Am. Jour. Med. Sci., April, 1867, p 333.

am rich, I am rich; I am worth ten thousand, a hundred thousand, a million of dollars;" and this insane idea increases until his plans would require unlimited means. Not as often, the insanity takes the form of political power; sometimes lasciviousness engrosses the man's thoughts, and he wishes to marry every woman whom he meets, and to beget countless children. This state of excitation is followed by depression, although the appetite and love for food, and the excessive venereal propensity continue. Sometimes, in the origin of the disorder, there was languor, pains in the head, excitability, and sleeplessness, and this condition was followed by great drowsiness; but in this secondary stage of the disease, these same symptoms are more marked, and paralytic changes commence with a modification of the speech, which consists in a want of coördination of the muscles of articulation, and very much resembles the attempts at conversation of a drunken man. This is accompanied by a twitching of the mouth, with tremulousness of the eyelids; the paralysis of the tongue becomes quite noticeable; slight paralytic strokes occur; sooner or later the patient has a maniacal paroxysm, sinks into dementia, and, after repeated epileptiform seizures, dies.

In grouping together the principal features of the autopsies made on patients dying of general paresis, we find, in most cases, that the cranium is thickened, the dura mater strongly adherent to the skull, and by filaments to the subjacent membranes; the arachnoid thickened, of a dark yellowish color, beneath it a considerable effusion of serum, and in several cases well-marked arachnoid cysts containing a red fluid or a firm blood coagulum; the pia mater usually congested; the brain engorged with blood or of a dusky hue, sometimes of a normal consistence, again with induration of the pons Varolii and olivary bodies, or with calcareous bodies in the pineal gland, but often with softening and degeneration of the brain substance. In one instance, there was fatty degeneration of the arteries of the brain, and a dark, slaty appearance of the spinal cord. These hints are enough to direct our attention to the necessity of the early diagnosis of this disease. B. D.

